# Neural network localization in Parkinson’s disease with impulse control disorders

**DOI:** 10.3389/fnagi.2025.1549589

**Published:** 2025-03-28

**Authors:** Hucheng Yang, Siyu Gu, Haihua Sun, Fengmei Zhang, Zhenyu Dai, Pinglei Pan

**Affiliations:** ^1^Department of Radiology, The Yancheng School of Clinical Medicine of Nanjing Medical University, Yancheng, China; ^2^Department of Radiology, Binhai Maternal and Child Health Hospital, Yancheng, China; ^3^Department of Neurology, The Yancheng School of Clinical Medicine of Nanjing Medical University, Yancheng, China

**Keywords:** Parkinson’s disease, impulse control disorders, network localization, voxel-based morphometry, functional connectivity network mapping

## Abstract

**Background:**

There is a huge heterogeneity of magnetic resonance imaging findings in Parkinson’s disease (PD) with impulse control disorders (ICDs) studies. Here, we hypothesized that brain regions identified by structural and functional imaging studies of PD with ICDs could be reconciled in a common network.

**Methods:**

In this study, an initial systematic literature review was conducted to collect and evaluate whole-brain functional and structural magnetic resonance imaging studies related to PD with ICDs. We subsequently utilized the Human Connectome Project (HCP) dataset (*n* = 1,093) and a novel functional connectivity network mapping (FCNM) technique to identify a common brain network affected in PD with ICDs.

**Results:**

A total of 19 studies with 25 contrasts, incorporating 345 individuals with PD and ICDs, and 787 individuals with PD without ICDs were included in the analysis. By using the HCP dataset and a novel FCNM technique, we ultimately identified that the aberrant neural networks predominantly involve the default mode network (middle and inferior temporal gyrus, anterior cingulate cortex, angular gyrus) and subcortical network (caudate nucleus).

**Conclusion:**

This study suggests that the heterogeneous neuroimaging findings in PD with ICDs can be attributed to shared abnormalities in the default mode and subcortical networks. These dysfunctions are associated with impaired self-regulation, decision-making, and heightened impulsivity in PD with ICDs. Our findings integrate diverse neuroimaging results from previous studies, providing a clearer understanding of the neurobiological mechanisms underlying PD with ICDs at a network level.

## 1 Introduction

Impulse control disorders (ICDs), characterized by compulsive behaviors such as pathological gambling, compulsive shopping, binge eating, and hypersexuality, are notably more prevalent in patients with Parkinson’s disease (PD) (Cilia and Van Eimeren, 2011; Weintraub et al., 2015; Voon et al., 2017; Antonini et al., 2017; Fantini et al., 2019). The prevalence of ICDs in the general population is estimated at 0.2–5.3%, but rises considerably to 20–46% among individuals with PD (Weintraub et al., 2015; Corvol et al., 2018). PD patients with ICDs often suffer from more exacerbated mental health problems correlated with the severity of their ICDs manifestations, resulting in notable personal, familial, and socioeconomic difficulties (Moegle et al., 2020; Dodd et al., 2005; Gan et al., 2021; Voon et al., 2011; Toś et al., 2023). The neural mechanisms underlying PD patients with ICDs are still not fully understood.

Multimodal magnetic resonance imaging (MRI) techniques have been applied to investigate brain functional and structural alterations in various neuropsychiatric diseases (Bowman et al., 2016; Long et al., 2012; Geranmayeh et al., 2014; Amemiya et al., 2024). Taking advantage of neuroimaging techniques, studies have discovered several brain regions that are related to PD with ICDs, with the inferior frontal gyrus, middle and inferior temporal gyri, anterior cingulate cortex (ACC), caudate nucleus, precentral gyrus, and angular gyrus being the relatively more affected (Gan et al., 2021; Esteban-Peñalba et al., 2021; Tessitore et al., 2017a; Maggi et al., 2024; Frosini et al., 2010). However, the findings exhibit significant heterogeneity, likely influenced by variations in study designs or analytical methods (Müller et al., 2018; Lin et al., 2023). Recent studies suggest that neuropsychiatric symptoms and diseases may be more accurately mapped to common brain networks rather than isolated regions (Kletenik et al., 2022; Boes et al., 2015; Cohen et al., 2019; Tetreault et al., 2020; Zhang et al., 2024). This network-based approach shifts the focus from isolated brain regions to a broader brain connectivity framework, benefiting from techniques like functional connectivity network mapping (FCNM) (Kletenik et al., 2022; Joutsa et al., 2022; Fox, 2018; Peng et al., 2022). This approach is conceptually similar to lesion network mapping, which uses lesion locations in patients to identify common networks associated with specific symptoms (Fox, 2018). By integrating neuroimaging data with large-scale brain connectome dataset, researchers can identify common symptom-specific networks underlying various neurological and psychiatric conditions (Kletenik et al., 2022; Boes et al., 2015; Kim et al., 2021; Wawrzyniak et al., 2018). Despite the extensive neuroimaging studies conducted on PD with ICDs, there is still limited research investigating network localization of PD with ICDs.

The objective of our research was to identify brain network disturbances associated with ICDs in PD by integrating results from diverse neuroimaging modalities. Our study utilized the Human Connectome Project (HCP) dataset due to its high-quality and large-scale resting-state functional MRI (fMRI) dataset, which can reduce individual variability and provide more reliable results in network mapping. Initially, we performed a systematic review of existing literature on structural and functional brain abnormalities in PD with ICDs. Next, leveraging large-scale resting-state fMRI dataset from the HCP dataset (Van Essen et al., 2013) and applying the FCNM approach (Mo et al., 2024) to distinct brain networks associated with ICDs in PD, which can enhance our understanding of the neural correlates and offer potential targets for therapeutic interventions.

## 2 Methods

### 2.1 Literature search and selection

Adhering to the Preferred Reporting Items for Systematic Reviews and Meta-analyses guidelines (Moher et al., 2009), a comprehensive systematic search was conducted in PubMed, Embase, and Web of Science to identify relevant studies published until July 30, 2024. We used specific keyword combinations to identify relevant articles: (“Parkinson’s disease” OR “Parkinson*”) AND (“impulsive control disorders” OR “pathological gambling” OR “hypersexuality” OR “compulsive eating” OR “compulsive shopping” OR “compulsive buying” OR “punding” OR “compulsive sexual behavior”) AND (“magnetic resonance imaging” OR “neuroimaging” OR “MRI” OR “resting state functional MRI” OR “rs-fMRI” OR “brain connectivity” OR “FC” OR “functional connectivity” OR “ReHo” OR “regional homogeneity” OR “ALFF” OR “amplitude of low frequency fluctuations” OR “fALFF” OR “fractional amplitude of low-frequency fluctuations” OR “cerebral blood flow” OR “CBF” OR “arterial spin labeling” OR “ASL” OR “independent component analysis” OR “ICA” OR “degree centrality” OR “DC” OR “PET” OR “positron emission tomography” OR “SPECT” OR “single photon emission computed tomography” OR “structural magnetic resonance imaging” OR “morphometry” OR “voxel-based” OR “voxel-wise” OR “voxel-based morphometry” OR “VBM” OR “high-resolution imaging” OR “structural neuroimaging” OR “DBM” OR “deformation-based morphometry” OR “GMV” OR “gray matter volume” OR “grey matter” OR “gray matter”). We systematically reviewed relevant meta-analyses and reviews to identify studies potentially omitted from our database search. A flow diagram of the study selection process is shown in Supplementary Figure S1. Studies were considered eligible based on the following inclusion criteria: (1) was published in an English-language peer-reviewed journal as an original article; (2) comparisons of gray matter volume (GMV), task-related activation, or resting-state activity between individuals with PD and ICDs and those with PD without ICDs; (3) analyses conducted voxel-wisely at the whole-brain level; (4) significant results either corrected for multiple comparisons or uncorrected; (5) significant brain cluster coordinates reported in standard reference space (Talairach or Montreal Neurological Institute space [MNI]). Exclusion criteria included: (1) no coordinate system reported; (2) use of region of interest (ROI) analysis: Analyses of local brain function, such as amplitude of low frequency fluctuations (ALFF) and regional homogeneity (ReHo), were conducted at the whole-brain level, without *a priori* hypotheses. Functional connectivity (FC) analysis was performed using a seed-based approach to investigate whole-brain connectivity patterns, explicitly excluding ROI-to-ROI analyses; (3) all reported coordinates outside the gray matter mask; (4) review or meta-analysis studies. Our analysis concentrated on contrasts rather than studies. Coordinates of peak voxels of significant clusters reported in each contrast were extracted, with coordinates in Talairach space converted to MNI space.

### 2.2 fMRI data acquisition and preprocessing

We employed the HCP 1200 Subjects Release (S1200) dataset, which contains resting-state fMRI data on healthy young adults 22–37 years of age. In our study, a total of 1,093 healthy adults [594 female; mean (SD) age 28.78 (3.69) years] were included. Exclusion criteria for this dataset included contraindications to MRI, current psychiatric or neurological disorders, use of psychiatric medications within the previous 3 months, pregnancy, and any history of head trauma. Detailed information about the sample is available in a separate publication (Peng et al., 2022). The demographic details of the HCP are presented in Supplementary Table S1.

The data from the HCP was collected using a 3 T Siemens Trio scanner, ensuring high-resolution imaging for comprehensive analyses. The resting-state fMRI parameters of the HCP dataset are provided in Supplementary Table S2. Participants exhibiting substandard image quality, including visible artifacts or incomplete brain coverage, were excluded from the analysis.

Resting-state fMRI data were preprocessed using SPM12 software^1^ and DPABI^2^ (Yan et al., 2016). The first 10 volumes for each participant were discarded to allow the signal to reach equilibrium and the participants to adapt to the scanning noise. The remaining volumes were corrected for the acquisition time delay between slices. Then, realignment was performed to correct the motion between time points. Head motion parameters were computed by estimating the translation in each direction and the angular rotation on each axis for each volume. All participant’s data were within the defined motion thresholds (i.e., maximal translational or rotational motion parameters <2 mm or 2°). We also calculated framewise displacement, which indexes the volume-to-volume changes in head position. Several nuisance covariates (linear drift, estimated motion parameters based on the Friston-24 model, spike volumes with framewise displacement >0.5 mm, global signal, white matter signal, and cerebrospinal fluid signal) were regressed out from the data. Because global signal regression can enhance the detection of system-specific correlations and improve the correspondence to anatomical connectivity (Murphy and Fox, 2017), we included this step in the preprocessing of resting-state fMRI data. Then, the datasets were bandpass filtered using a frequency range of 0.01–0.1 Hz. In the normalization step, individual structural images were first coregistered with the mean functional images; the transformed structural images were then segmented and normalized to MNI space using a high-level nonlinear warping algorithm, i.e., the diffeomorphic anatomical registration through the exponentiated Lie algebra technique (Murphy and Fox, 2017). Next, each filtered functional volume was spatially normalized to MNI space using the deformation parameters estimated during the above step and resampled into a 3-mm isotropic voxel. Finally, all data were spatially smoothed with a Gaussian kernel of 6 × 6 × 6 mm^3^ full width at half maximum.

### 2.3 Functional connectivity network mapping

The FCNM method was utilized for the construction of networks related to PD with ICDs, delineating brain structural and functional dysfunctional networks by analyzing GMV, task-induced activation, and resting-state activity differences between PD with ICDs and without ICDs. First, 4-mm radius spheres were created at the coordinates of each contrast and merged to form a contrast-specific seed mask, referred to as the contrast seed. Next, using preprocessed resting-state fMRI data from the HCP, we generated a contrast seed to whole brain FC map for each participant by calculating Pearson’s correlation coefficients between the time courses of the contrast seed and every voxel across the brain. These correlation values were then subjected to Fisher’s z-transformation to normalize the data. Subsequently, the 1,093 subject-level FC maps were entered into a voxel wise one-sample *t*-test to identify brain regions functionally connected to each contrast seed. Only positive FC values were considered, as the interpretation of negative FC remains controversial (Murphy and Fox, 2017). Group-level t-maps were thresholded and binarized at *p* < 0.05, corrected for multiple comparisons using the false discovery rate (FDR) method. Finally, binarized maps of gray matter volume, task-induced activation, and resting-state activity contrasts were overlaid to generate network probability maps, which were thresholded at 60% based on previous well-validated FCNM studies (Zhang et al., 2024; Mo et al., 2024) to define the PD with ICDs task activation, resting-state activity and GMV dysfunctional networks.

### 2.4 Association with canonical brain networks

To improve interpretability, we examined the spatial relationships PD with ICDs brain dysfunctional networks and 8 well-recognized canonical brain networks. The seven cortical networks were defined as the visual, somatomotor, dorsal attention, ventral attention, limbic, frontoparietal, and default networks according to the Yeo et al. (2011) study. The Human Brainnetome Atlas was utilized to delineate the subcortical network (Fan et al., 2016), encompassing the amygdala, hippocampus, basal ganglia, and thalamus. The spatial association was measured by determining the proportion of overlapping voxels between each PD and ICDs brain dysfunctional network and the corresponding canonical network, normalized by the total voxel count within the canonical network.

## 3 Results

### 3.1 Included studies and sample characteristics

Initially, a total of 1,075 relevant documents were identified and then underwent a rigorous screening process; and 19 studies with 25 contrasts, incorporating data from 345 individuals with PD and ICDs, along with 787 individuals with PD without ICDs were finally included in the analysis. These included studies covered a variety of ICDs, including pathological gambling, compulsive shopping, compulsive eating, hypersexuality, binge eating, and punding. This research encompassed 17 functional studies (6 rs-fMRI studies, 5 task-based fMRI studies, 4 PET studies, and 3 SPECT studies) and 2 VBM studies. Details regarding the sample and imaging characteristics of the included studies are summarized in Table 1.

**Table 1:** Sample and imaging characteristics of the studies included in the analysis.

Study	*N* (PD-ICDs/PD)	Mean age (SD)	Gender (male)	UPDRS-score	ICDs type	Cognitive status	Imaging modality	Method	Software	Threshold
Cilia et al. (2008)	11/40	57.4 (5.8)	90.9%	UPDRS-III 18 (11)	PG	MMSE: 28.9 (0.8)	SPECT	rCBF	SPM2	*P* ≤ 0.05 (FDR)
Frosini et al. (2010)	7/7	57.5 ± 11	NA	UPDRS-III 15.5 ± 1.3	PG	MMSE: 29.6	Task fMRI	BOLD	NA	*p* ≤ 0.01 (FDR)
Rao et al. (2010)	9/9	56.2 (10.9)	77.8%	NA	PG; CE; CS; BE	MoCA: 27. 1 (1.8)	Task fMRI rs-fMRI	rCBF	SPM2	*P* ≤ 0.05 (FDR)
van Eimeren et al. (2009)	7/7	60 (9.8)	100%	NA	PG	MoCA: 27.1 (2.5)	PET	rCBF	SPM5	NA
Voon et al. (2011)	14/14	51.5 (8.3)	71.4%	NA	PG	MMSE: 27.7 (3. 12)	Task fMRI	BOLD	SPM5	NA
Ray et al. (2012)	7/7	59.7 (10.9)	100%	UPDRS-III 21 (8.04)	PG	NA	PET	rCBF	SPM8	*P* ≤ 0.05 (FDR)
Lee et al. (2014)	11/11	56.7 (8.7)	72.7%	UPDRS-III 14.2 (11)	HS; PG; CE; CS; punding	MMSE: 27.7 (1.6)	PET	rCBF	SPM8	NA
Premi et al. (2016)	21/63	65.8 (8.4)	23.8%	UPDRS-III 16.5 (7.2)	CE; PG; HS; punding	MMSE: 27.7 (1.9)	SPECT	rCBF	SPM8	*p* < 0.005
Cilia et al. (2008)	15/15	59.2 (7.6)	93.3%	UPDRS-III 16.9 (8.8)	PG	MMSE: 28.6 (0.9)	SPECT	rCBF	SPM8	*P* < 0.05
Tessitore et al. (2017a)	15/15	57 (9.7)	33.3%	UPDRS-III 15.7 (6)	HS; CE; PG; CS	MMSE:28.8 (8.6) MoCA:24.6 (4. 1)	rs-fMRI	FC	SPM12	NA
Tessitore et al. (2017b)	15/15	62.87 (8.6)	86.6%	UPDRS-III 10.9 (4.5)	HS; CE; PG; punding	MMSE: 26.5 (2.2)	rs-fMRI	FC	SPM8	*P* ≤ 0.05 (FWE)
Filip et al. (2018)	8/13	65 (5.7)	75%	NA	ICD	NA	Task fMRI	BOLD	SPM12	*P* ≤ 0.05 (FWE)
Verger et al. (2018)	18/18	60.4 (67.3)	83.3%	UPDRS-III off 31.9 (12) UPDRS-III on 5.3 (4.1)	PG; HS; CS	NA	PET	rCBF	SPM8	*P* < 0.005
Esteban-Peñalba et al. (2021)	18/17	63.33 (8.24)	88.9%	UPDRS-III 21.5[10–46]	ICD	NA	Task fMRI	BOLD	SPM8	*P* ≤ 0.05 (FWE)
Gan et al. (2021)	21/33	59 ± 9.6	57.1%	UPDRS-III 20.3 ± 14.2	PG; HS; BE; CS	MMSE: 28.3 ± 1. 1	rs-fMRI	VMHC	SPM8	*p* < 0.01
Kimura et al. (2023)	37/37	66.0 (11.6)	46%	NA	ICD	MMSE: 26.7 (3.9)	rs-fMRI	FC	SPM12	*P* < 0.05
Maggi et al. (2024)	65/287	60.23 ± 10.45	63%	NA	ICB	MoCA:26.69 ± 2.49	Structural MRI	VBM	SPM12	*P* ≤ 0.05 (FWE)
Baagil et al. (2023)	36/36	65.7 (9.0)	66.6%	UPDRS-III 20.3 (14. 1)	ICB	MoCA: 26.7 (3.7)	Structural MRI	VBM	SPM12	*P* ≤ 0.05 (FWE)
Theis et al. (2022)	10/10	56.2 (12.37)	80%	UPDRS-III 22.56 (11.48)	HS	MoCA: 27.7 (1.5)	rs-fMRI	BOLD	SPM8	*p* < 0.001

PD, Parkinson’s disease; ICDs, impulse control disorders; BOLD, blood oxygen level dependent; FC, functional connectivity; VMHC, voxel-mirrored homotopic connectivity; FDR, false discovery rate; FWE, family-wise error; MMSE, Mini-Mental State Examination; MoCA, Montreal Cognitive Assessment; N, sample size; NA, not applicable; PET, positron emission tomography; rCBF, regional cerebral blood flow; rs-fMRI, resting-state functional magnetic resonance imaging; SPECT, single photon emission computed tomography; UPDRS, Unified Parkinson’s Disease Rating Scale; VBM, voxel-based morphometry; SPM, Statistical Parametric Mapping; PG, pathological gambling; CE, compulsive eating; CS, compulsive buying; HS, hypersexuality; BE, binge-eating; ICB: impulse control behaviors.

### 3.2 Abnormal brain networks associated with ICDs in PD

**Figure 1:**
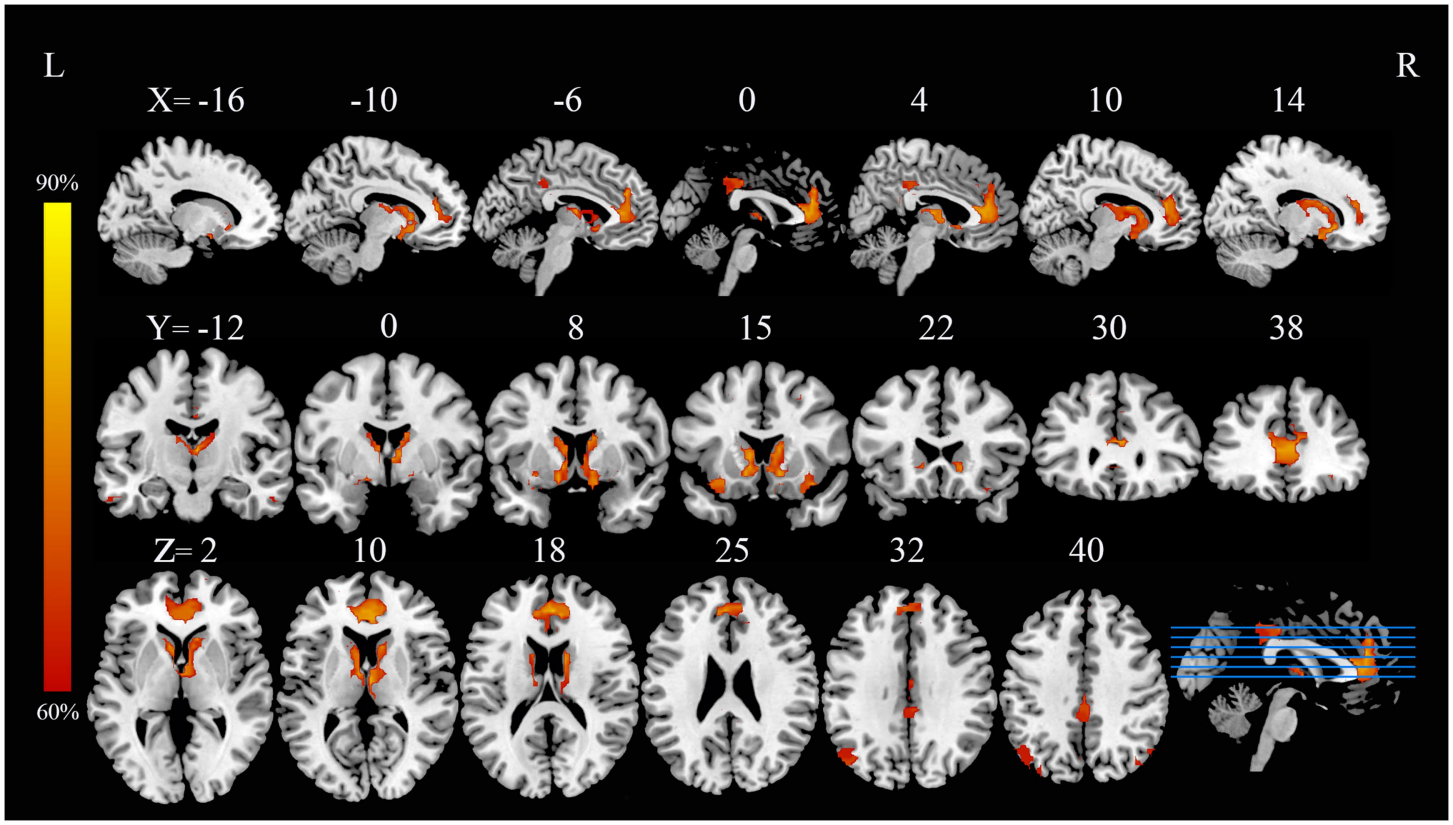
PD with ICDs brain dysfunctional networks based on 4-mm radius sphere dysfunctional networks are shown as network probability maps thresholded at 60%, showing brain regions functionally connected to more than 60% of the contrast seeds. PD with ICDs, Parkinson’s disease with impulse control disorders.

**Figure 2:**
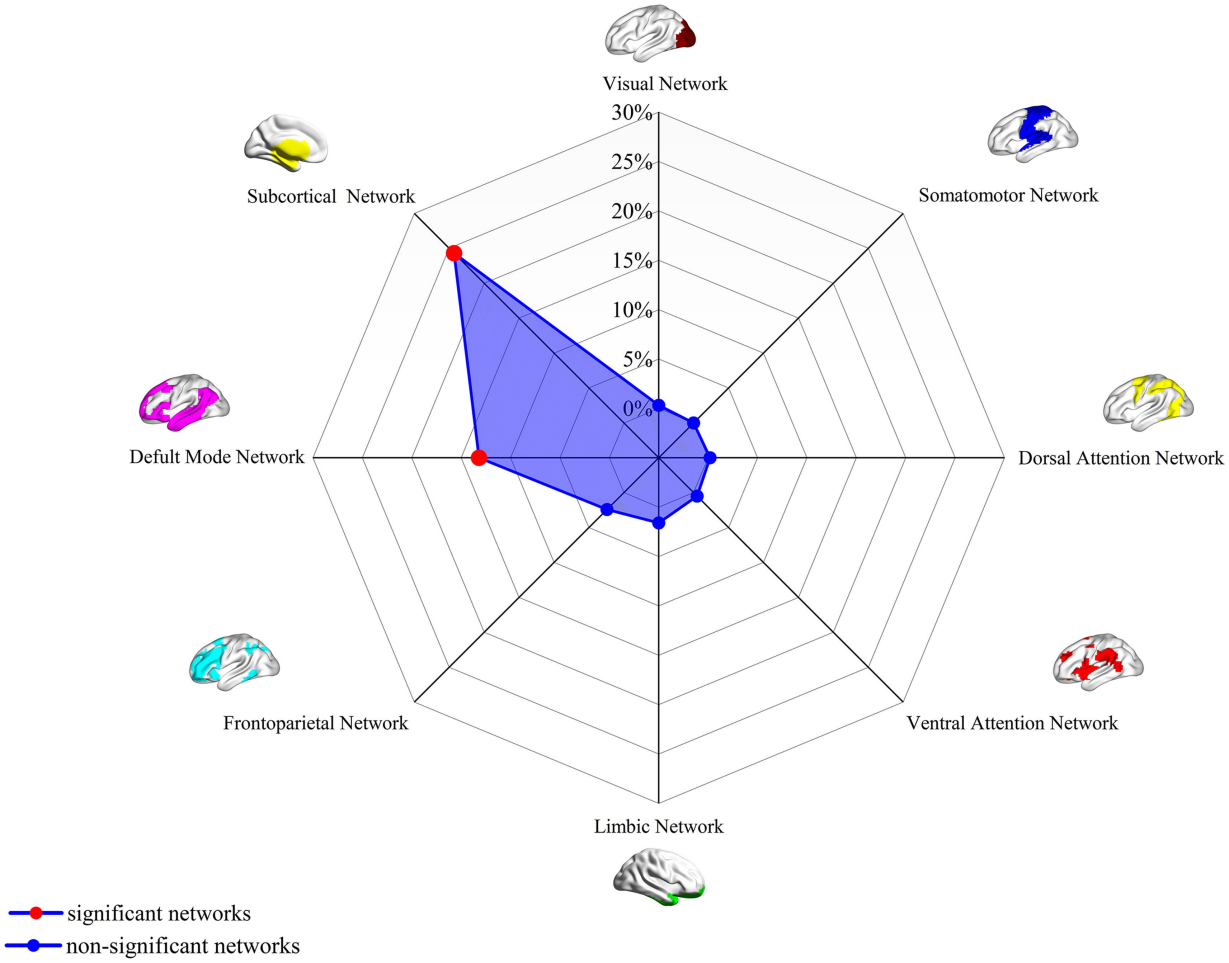
Associations of dysfunctional brain networks with canonical brain networks in PD with ICDs based on 4-mm radius sphere. Polar plots display the proportion of overlapping voxels between each brain dysfunctional network and a canonical network relative to all voxels within the corresponding canonical network. The red circles represent brain dysfunction networks, defined as significant networks, exhibiting ≥10% overlap with canonical networks, whereas the blue circles represent non-significant networks with <10% overlap. PD with ICDs, Parkinson’s disease with impulse control disorders.

In current study, using the HCP dataset and FCNM technique, we found that the disrupted neural networks predominantly involve the DMN and subcortical network, with specific brain nodes: the middle and inferior temporal gyri, ACC, angular gyrus, and caudate nucleus (Figure 1). Additionally, our study revealed that the results obtained using the 1-mm (Supplementary Figure S2) and 7-mm (Supplementary Figure S3) radii closely mirrored those from the 4-mm radius when the FCMN procedure was repeated. The DMN includes regions such as the middle and inferior temporal gyri, ACC, and angular gyrus, with an overlap proportion of 13.2%. The subcortical network primarily includes the caudate nucleus, with an overlap proportion of 24.1% (Figure 2). The overlap proportions with other brain networks, such as visual network, somatomotor network, dorsal attention network, ventral attention network, limbic network, and frontoparietal network, were all <10%. Subsequently, replicating the FCNM procedure with spheres of 1-mm (Supplementary Figure S4) and 7-mm (Supplementary Figure S5) radii, the resulting brain dysfunctional networks closely mirrored those obtained using the 4-mm radius sphere.

## 4 Discussion

To the best of our knowledge, the current study is the first to use the FCMN method to analyze abnormal brain networks in PD with ICDs. Through the innovative FCNM approach, we analyzed data from 19 studies with 25 contrasts examining a sample of 345 PD patients with ICDs and 787 patients without ICDs, identifying distinct brain networks associated with PD with ICDs across multiple imaging modalities. Our findings revealed that abnormal brain networks mainly involved the DMN, including regions such as the middle and inferior temporal gyri, ACC, and angular gyrus, along with the subcortical network, particularly the caudate nucleus. Network overlap was calculated using a 4-mm radius sphere, and validations with sphere radii of 1 and 7-mm yielded robust and replicable results. These findings indicate that the DMN and subcortical network likely related to the pathogenesis and maintenance of ICDs, offering promising avenues for forthcoming therapeutic strategies.

### 4.1 Abnormal DMN in PD with ICDs

The DMN is crucial for self-referential processes, social cognition, autobiographical memory, and prospective imagery (Spreng et al., 2009; Wang et al., 2017). It comprises the medial prefrontal cortex, middle and inferior temporal gyri, posterior cingulate cortex, ACC, precuneus, and angular gyrus (Liao et al., 2011; Davey et al., 2016). Studies have indicated that individuals with PD who also exhibit ICDs show disrupted connectivity within the DMN, which may be related to patients focusing more intensely on internal impulses and thoughts, thereby aggravating the symptoms of ICDs (Ma et al., 2011; Morese and Palermo, 2020). Furthermore, altered FC between the DMN, control network, and dorsal attention network may be a key factor contributing to impulsive behavior, making suppression of such behaviors more challenging (Ma et al., 2011; Zhu et al., 2021). Additionally, PD patients with ICDs struggle to effectively suppress DMN activity during task execution, which are believed to contribute to poor decision-making and more prone to impulsive behaviors (Tessitore et al., 2017b; Meyer et al., 2019). Disrupted DMN connectivity in PD patients with ICDs can impair self-referential processing and self-monitoring, reducing their capacity to regulate internal impulses and promoting impulsive behaviors (Tessitore et al., 2017b; van Eimeren et al., 2009). DMN hyperactivity may indirectly impair self-monitoring capacity by suppressing the executive control network, thereby diverting attention and fostering competition for cognitive resources (Christoff et al., 2009; Sonuga-Barke and Castellanos, 2007). Repetitive transcranial magnetic stimulation (rTMS) has emerged in recent years as a promising non-invasive therapeutic modality for addressing both motor and non-motor symptoms in PD (Wagle Shukla et al., 2016; Aftanas et al., 2020; Brys et al., 2016). Clinical investigations indicate that rTMS targeting prefrontal cortical regions, notably the dorsolateral prefrontal cortex and orbitofrontal cortex, holds promise in mitigating the clinical manifestations of ICDs (Aftanas et al., 2020). Further research has demonstrated that rTMS can elicit improvements in specific cognitive domains in PD with ICDs, notably in areas such as inhibitory control, decision-making, and working memory (Grassi et al., 2021). During dopaminergic treatment in PD patients, aberrant FC across the three networks (DMN, central executive network, and the salience network) linked to the onset of ICDs (Tessitore et al., 2017a; Roussakis et al., 2019; Staubo et al., 2024). The pathological alterations in PD may underlie the emergence of ICDs, with dopamine treatment being intricately linked to both the onset and severity of ICDs (Staubo et al., 2024; Latella et al., 2019).

The middle and inferior temporal gyri play fundamental roles in object recognition, memory integration, language processing, and social cognition, positioning them as key regions for both visual perception and complex cognitive functions (Yeo et al., 2011; Huang et al., 2023; Conway, 2018). Several studies have indicated a significant association between the middle and inferior temporal gyri and ICDs in PD (Roussakis et al., 2019; Carriere et al., 2015; Marques et al., 2021; Zsidó et al., 2019). Structural and functional imaging studies revealed that disruptions in the corticostriatal pathways involving the middle and inferior temporal gyri related to impaired reward processing and ICDs in PD (Roussakis et al., 2019; Carriere et al., 2015; Aracil-Bolaños and Strafella, 2016). Specifically, the middle and inferior temporal gyri are linked to dysfunctional connectivity with regions such as the ACC and ventral striatum, which are central to decision-making and risk evaluation (Marques et al., 2021; Zsidó et al., 2019). These abnormalities are considered fundamental to the emergence of impulsive and compulsive behaviors, such as pathological gambling and hypersexuality, seen in PD patients (Roussakis et al., 2019; Marques et al., 2021).

The ACC is a complex region crucial for emotional regulation, cognitive control, error detection, and autonomic functions, serving as a key integrator of emotional and cognitive processes (Alexopoulos et al., 2008; Reimann et al., 2023; Etkin et al., 2011). Dysfunction in the ACC is linked to heightened impulsivity and diminished cognitive control (Reimann et al., 2023; Etkin et al., 2011). For example, the research conducted by Santangelo et al. (2019) highlighted a notable decrease in functional connectivity within the ACC, indicating a correlation with compromised impulse inhibition (Santangelo et al., 2019). Further research indicates that impairments in the ACC, combined with ventral striatum damage, related to the development of ICDs (Carriere et al., 2015; Paz-Alonso et al., 2020; Probst and van Eimeren, 2013; Filip et al., 2018). In the context of addictive disorders, characterized by heightened impulsivity and compulsive behaviors, the ACC is recognized as a key region involved in regulating these impulsive actions (Goldstein and Volkow, 2011; Koob and Volkow, 2016). After TMS treatment in PD with ICDs, it was observed that the treatment could possibly enhance FC between the ACC and various brain regions, especially the prefrontal cortex and limbic system, which may improve emotional regulation and impulse control (Qiu et al., 2023; Balderston et al., 2022). On the other hand, TMS seems to facilitate neuroplasticity in the ACC, suggesting a potential restoration of its functional role in impulse control and an alleviation of impulsive symptoms (Zhang et al., 2021; Vitale et al., 2019).

The angular gyrus integrates multisensory inputs, facilitating semantic processing, episodic memory, and the creation of autobiographical narratives (Milardi et al., 2019; Ji et al., 2019). The angular gyrus, as part of the parietal cortex, processes sensory and motor inputs, making it integral to tasks requiring impulse regulation (Ji et al., 2019; Seghier, 2013; De Micco et al., 2018). Dysfunction of the angular gyrus may result in disturbances within the prefrontal-striatal and midbrain-limbic systems, which are linked to the development of ICDs in PD (Filip et al., 2018; Seghier, 2023). Additionally, dopamine replacement therapy may induce and exacerbate the occurrence of ICD symptoms, which seems to be linked to the overstimulation of the dopamine system in reward pathways, with involvement of the angular gyrus (Cilia and Van Eimeren, 2011; Dalley et al., 2011).

Taken together, our study suggests that ICDs are closely linked to dysfunctional DMN, particularly involving the middle and inferior temporal gyri, ACC, and angular gyrus. These brain regions and the corresponding DMN are associated with self-monitoring and self-regulation, which are significantly involved in the neural mechanisms developing ICDs in PD.

### 4.2 Abnormal subcortical network in PD with ICDs

The subcortical network is widely distributed and primarily includes the thalamus, caudate nucleus, hippocampus (Milardi et al., 2019; Ji et al., 2019). The subcortical network facilitates essential functions like movement control, emotional regulation, memory processing, and sensory integration, playing a pivotal role in both voluntary actions and automatic bodily functions (Milardi et al., 2019; Zhang et al., 2024; Šimić et al., 2021; Zilverstand et al., 2018). Recently studies have shown that the FC within the subcortical network of PD patients with ICDs undergoes significant alterations (Gan et al., 2021; Esteban-Peñalba et al., 2021; Petersen et al., 2018).

The caudate nucleus was identified as an important node in the subcortical network in patients with PD with ICDs in our study. The caudate nucleus, a critical component of the basal ganglia, is fundamentally involved in regulating a range of physiological processes, including motor control, learning, and higher cognitive functions (Zhang et al., 2024; Šimić et al., 2021; Grahn et al., 2009). ICDs in PD patients have been found to be connected to aberrant FC the caudate nucleus, possibly involving brain regions associated with emotion, cognition, and sensation (Zilverstand et al., 2018; Hepp et al., 2017). Neuroimaging research shows that dopamine agonists like pramipexole heighten impulsivity by influencing the anterior caudate, which governs impulsive choices (Lee et al., 2014; Martinez et al., 2020; Williams and Potenza, 2008). Moreover, the inhibitory control between the prefrontal cortex and the caudate nucleus is weakened in these patients, related to impulsive decision-making and behaviors (De Micco et al., 2018). Disruption of the regulatory balance within the subcortical–cortical circuits is believed to be a key factor of the emergence of ICDs in PD (Kelly et al., 2020). Impairments in the connectivity between the caudate and cortical regions further drive impulsive behaviors (De Simoni et al., 2018). Additionally, lower dopamine transporter binding in the caudate is linked to greater impulsivity, suggesting its role in controlling inhibitory actions (Smith et al., 2018; London, 2020; Bu et al., 2021).

Ultimately, much like the DMN, the subcortical network is integral to the development of ICDs in PD. The abnormality of the caudate nucleus may disrupt neural circuits responsible for regulating impulsivity, which is associated with ICDs. Dysfunctions in subcortical network may relate to the dysregulation of inhibitory control and increased impulsivity, which making patients prone to ICDs like pathological gambling, hypersexuality, and compulsive shopping (Meyer et al., 2020).

### 4.3 Limitations and future perspectives

There are several limitations to this study. First, to identify brain network localization of PD with ICDs, we utilized resting-state fMRI data from a large sample of healthy adults provided by the HCP dataset. Since the HCP dataset consists of young healthy adults, it may not comprehensively reflect the network alterations associated with PD and ICDs, which could arise from aging, disease progression, or pharmacological interventions. It might be more suitable to use data more closely matching the demographic and clinical profiles of the patients featured in the selected studies. Second, given the limitations of previous research, we utilized multimodal neuroimaging data rather than a single modality, each with distinct physiological significance, which may have influenced the results; future studies employing single modalities separately could enhance accuracy in network localization. Third, Although the FCNM approach effectively identified common networks associated with PD and ICDs, subtle variations in network dysfunction may exist among different ICDs subtypes. Future studies with larger sample sizes and more detailed clinical phenotyping could investigate potential subtype-specific network signatures. Fourth, it is crucial to emphasize that our findings reveal a correlational relationship between the identified brain networks and this disorder. While these networks likely contribute to understand the pathophysiology of ICDs, our research is unable to determine the causal relationship between the current disease and the dysfunctional networks. Future longitudinal studies are needed to clarify the causal relationship between the current disease and network changes. Finally, drawing on the study discoveries regarding abnormal brain networks, upcoming research could integrate brain stimulation methods such as TMS or transcranial direct current stimulation to specifically address and regulate the deviant connections within the DMN and subcortical networks. This approach is designed to alleviate symptoms linked to ICDs.

## 5 Conclusion

In conclusion, our study utilized the novel FCNM approach with large-scale human brain connectome data to pinpoint brain structural and functional heterogeneity within brain functional networks in patients with ICDs in PD. Our study identified specific brain networks associated with ICDs symptoms, predominantly the DMN, which encompasses brain regions like the middle and inferior temporal gyri, ACC, and angular gyrus, and the subcortical network, which involves the caudate nucleus. These findings may help resolve inconsistencies in previous neuroimaging studies and enhance our understanding of the neurobiological mechanisms underlying PD with ICDs from a network-level perspective.

## Data Availability

The raw data supporting the conclusions of this article will be made available by the authors, without undue reservation.

## Footnotes

^1^ https://www.fil.ion.ucl.ac.uk/spm/

^2^ https://rfmri.org/DPABI

## Supplementary material

Supplementary Figure S1 A flow diagram of the study selection process. Analyses of local brain function, such as ALFF and ReHo, were conducted at the whole-brain level, without a priori hypotheses. FC analysis was performed using a seed-based approach to investigate whole-brain connectivity patterns, explicitly excluding ROI-to-ROI analyses. ALFF, amplitude of low-frequency fluctuations; FC, functional connectivity; GM, gray matter; PD with ICDs, Parkinson's disease with impulse control disorders; ReHo, regional homogeneity; ROI, region of interest VBM, voxel-based morphometry.

Supplementary Figure S2 PD with ICDs brain dysfunctional networks based on 1-mm radius sphere. Dysfunctional networks are shown as network probability maps thresholded at 60%, showing brain regions functionally connected to more than 60% of the contrast seeds. PD with ICDs, Parkinson's disease with impulse control disorders.

Supplementary Figure S3 PD with ICDs brain dysfunctional networks based on 7-mm radius sphere. Dysfunctional networks are shown as network probability maps thresholded at 60%, showing brain regions functionally connected to more than 60% of the contrast seeds. PD with ICDs, Parkinson's disease with impulse control disorders.

Supplementary Figure S4 Associations of dysfunctional brain networks with canonical brain networks in PD with ICDs based on 1-mm radius sphere. Polar plots display the proportion of overlapping voxels between each brain dysfunctional network and a canonical network relative to all voxels within the corresponding canonical network. The red circles represent brain dysfunction networks, defined as significant networks, exhibiting ≥ 10% overlap with canonical networks, whereas the blue circles represent non-significant networks with <10% overlap. PD with ICDs, Parkinson's disease with impulse control disorders.

Supplementary Figure S5 Associations of dysfunctional brain networks with canonical brain networks in PD with ICDs based on 7-mm radius sphere. Polar plots display the proportion of overlapping voxels between each brain dysfunctional network and a canonical network relative to all voxels within the corresponding canonical network. The red circles represent brain dysfunction networks, defined as significant networks, exhibiting ≥ 10% overlap with canonical networks, whereas the blue circles represent non-significant networks with < 10% overlap. PD with ICDs, Parkinson's disease with impulse control disorders.

## Glossary

ACC: anterior cingulate cortex
ALFF: amplitude of low-frequency fluctuation
ASL: arterial spin labeling
BE: binge-eating
BOLD: blood-oxygen-level-dependent
CBF: cerebral blood flow
CE: compulsive eating
CS: compulsive buying
DAN: dorsal attention network
DBM: deformation-based morphometry
DC: degree centrality
DMN: default mode network
DPABI: Data Processing and Analysis of Brain Imaging
FA: flip angle
fALFF: fractional amplitude of low-frequency fluctuation
FC: functional connectivity
FCNM: functional connectivity network mapping
fMRI: functional MRI
FDR: false discovery rate
FWE: family-wise error
FOV: field of view
GRE-EPI: gradient-recalled echo-planar imaging
GMV: gray matter volume
HS: hypersexuality
ICA: independent component analysis
ICB: Impulse control behaviors
ICDs: impulse control disorders
MRI: magnetic resonance imaging
MMSE: Mini-Mental State Examination
MoCA: Montreal Cognitive Assessment
NA: not applicable
PG: pathological gambling
PD: Parkinson’s disease
PET: positron emission tomography
ROI: region of interest
rTMS: repetitive transcranial magnetic stimulation
rs-fMRI: resting-state functional magnetic resonance imaging
ReHo: regional homogeneity
rCBF: regional cerebral blood flow
SPECT: single photon emission computed tomography
SPM: Statistical Parametric Mapping
TE: echo time
TR: repetition time
UPDRS: Unified Parkinson’s disease rating scale
VBM: voxel-based morphometry
VMHC: voxel-mirrored homotopic connectivity
