# Supplementary figures and images for: Neural network localization in Parkinson’s disease with impulse control disorders
Source: Front Aging Neurosci. 2025 Mar 28;17:1549589. doi: 10.3389/fnagi.2025.1549589 (PMC11985847; doi:10.3389/fnagi.2025.1549589)

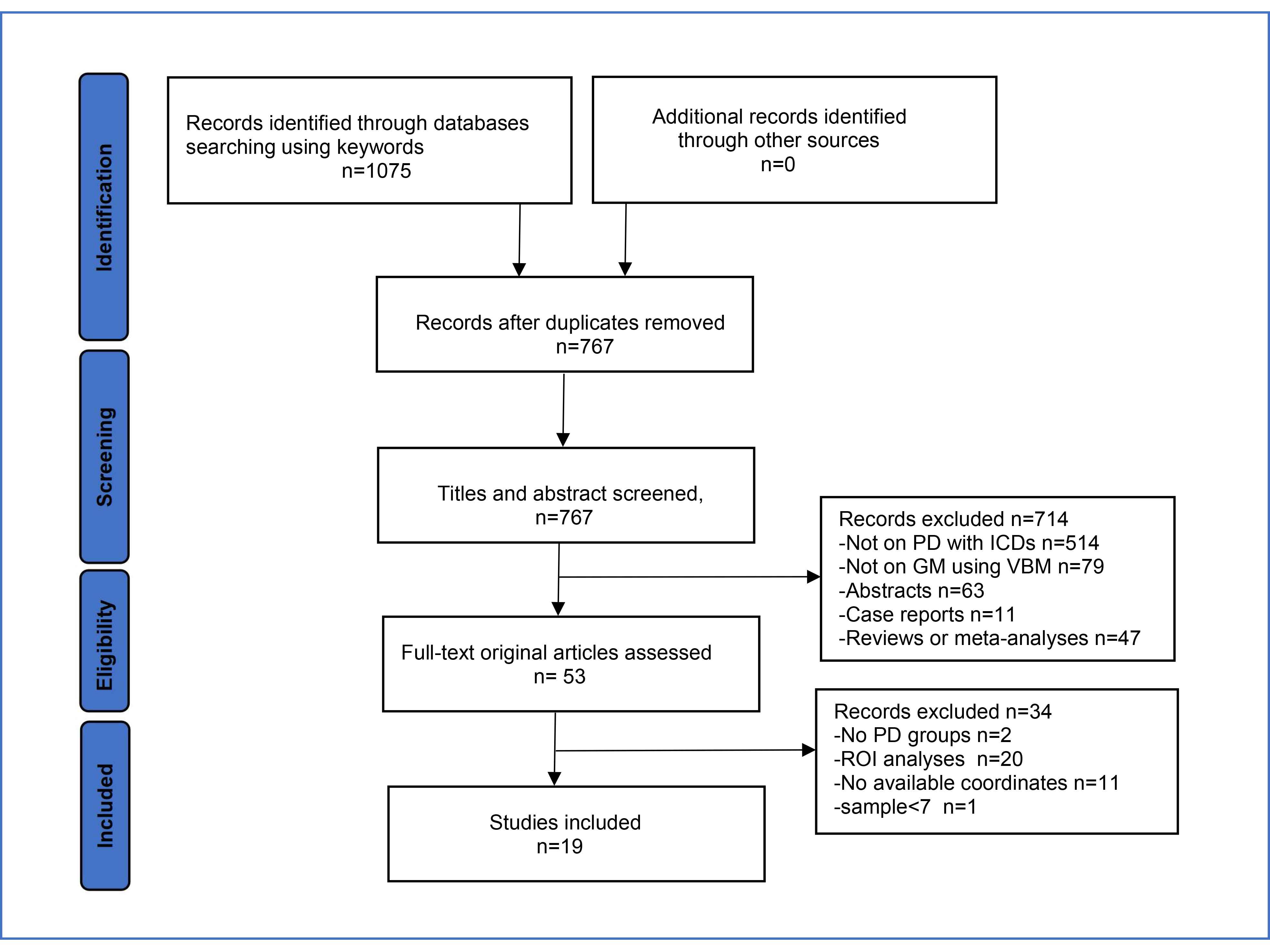

Supplement: Supplementary Figure S1 — A flow diagram of the study selection process. Analyses of local brain function, such as ALFF and ReHo, were conducted at the whole-brain level, without a priori hypotheses. FC analysis was performed using a seed-based approach to investigate whole-brain connectivity patterns, explicitly excluding ROI-to-ROI analyses. ALFF, amplitude of low-frequency fluctuations; FC, functional connectivity; GM, gray matter; PD with ICDs, Parkinson's disease with impulse control disorders; ReHo, regional homogeneity; ROI, region of interest VBM, voxel-based morphometry. [file Image_1.tif]

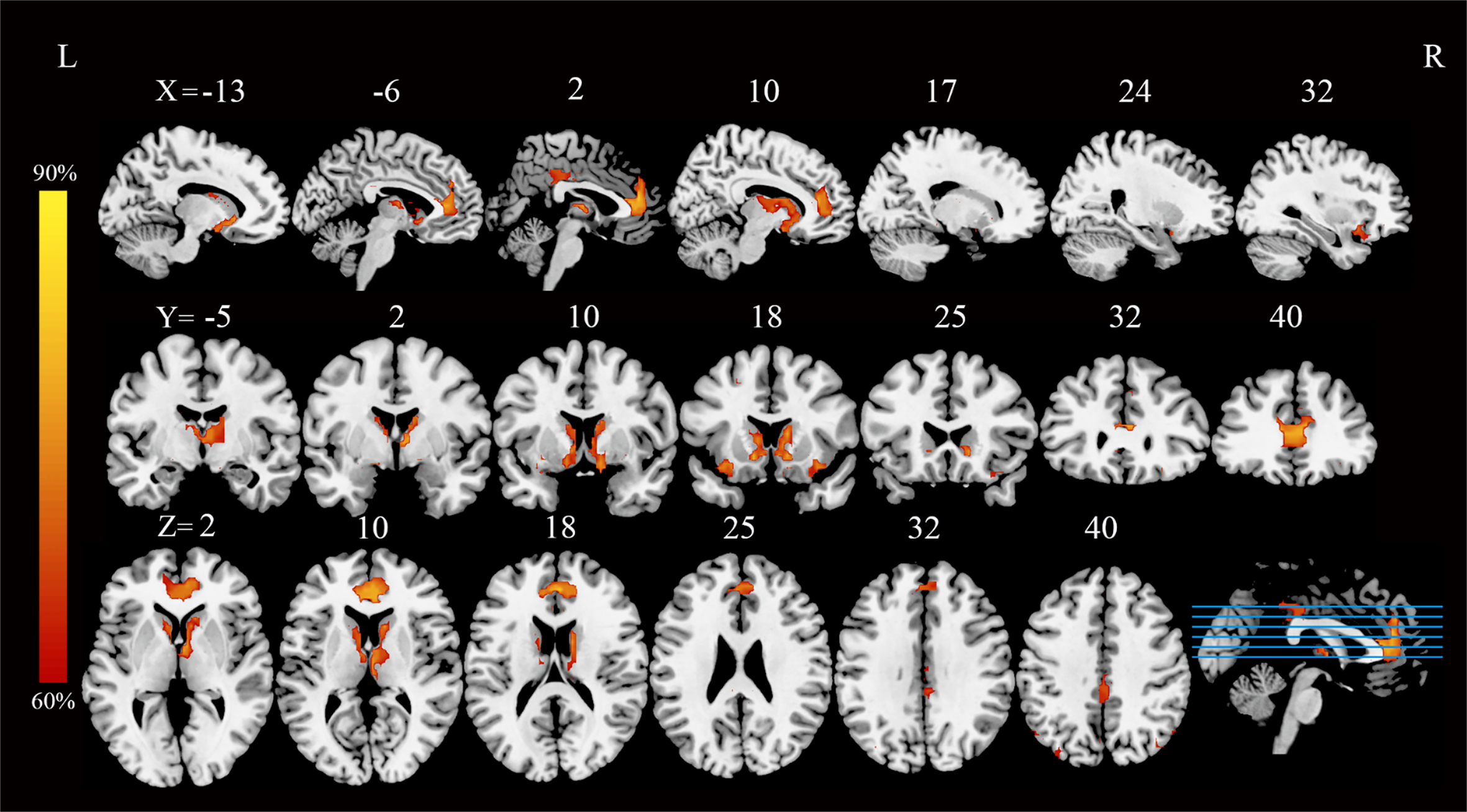

Supplement: Supplementary Figure S2 — PD with ICDs brain dysfunctional networks based on 1-mm radius sphere. Dysfunctional networks are shown as network probability maps thresholded at 60%, showing brain regions functionally connected to more than 60% of the contrast seeds. PD with ICDs, Parkinson's disease with impulse control disorders. [file Image_2.tif]

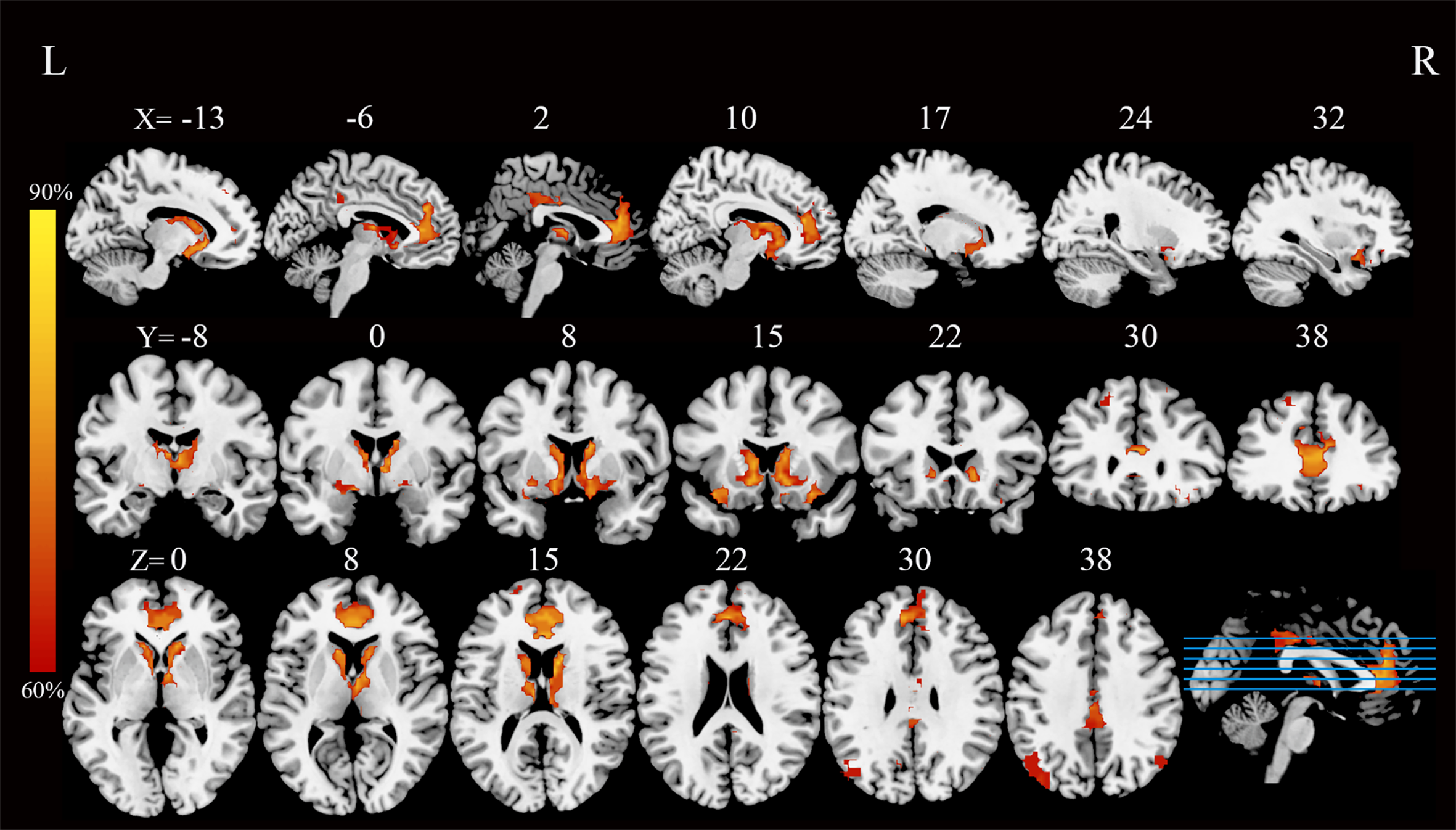

Supplement: Supplementary Figure S3 — PD with ICDs brain dysfunctional networks based on 7-mm radius sphere. Dysfunctional networks are shown as network probability maps thresholded at 60%, showing brain regions functionally connected to more than 60% of the contrast seeds. PD with ICDs, Parkinson's disease with impulse control disorders. [file Image_3.tif]

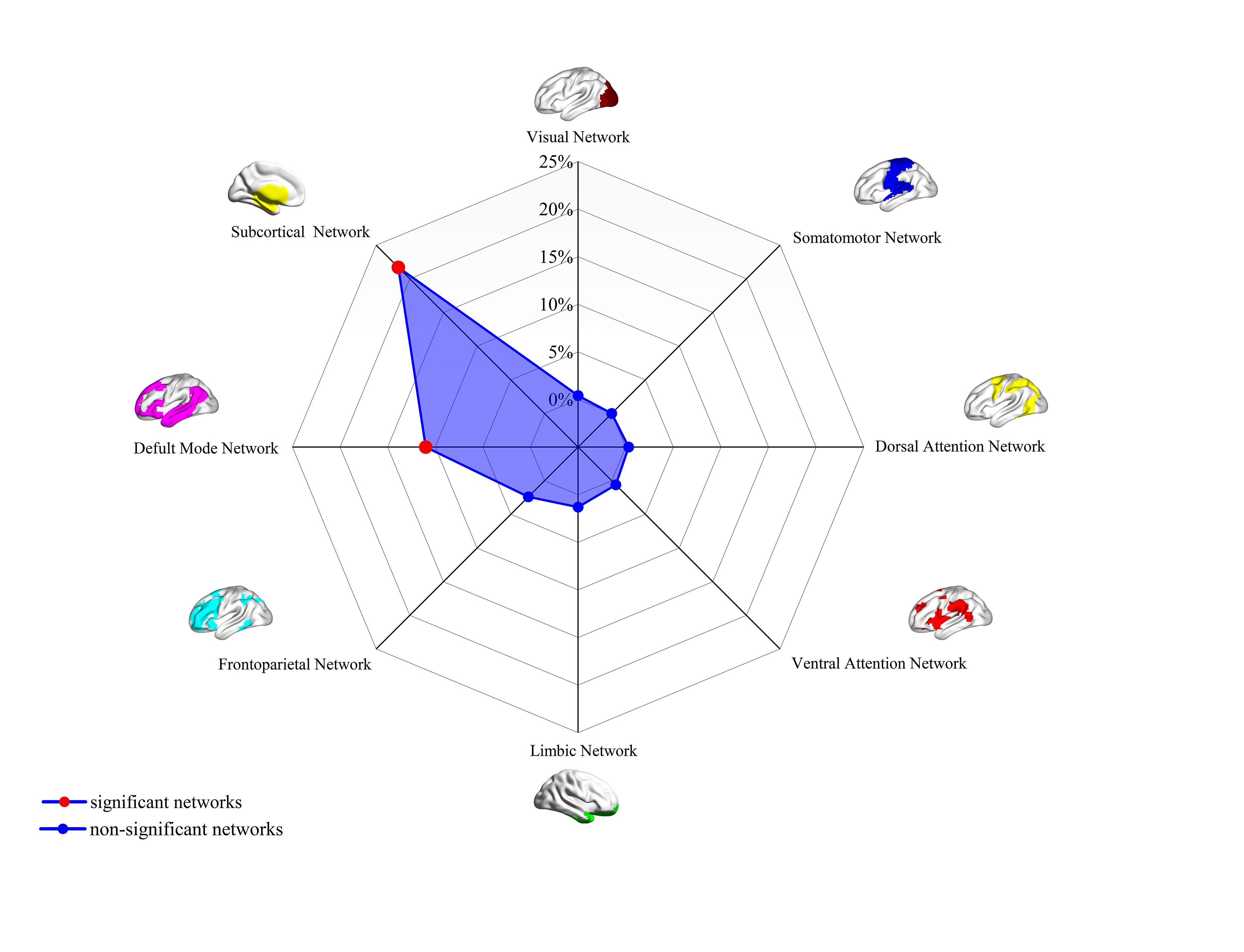

Supplement: Supplementary Figure S4 — Associations of dysfunctional brain networks with canonical brain networks in PD with ICDs based on 1-mm radius sphere. Polar plots display the proportion of overlapping voxels between each brain dysfunctional network and a canonical network relative to all voxels within the corresponding canonical network. The red circles represent brain dysfunction networks, defined as significant networks, exhibiting ≥ 10% overlap with canonical networks, whereas the blue circles represent non-significant networks with <10% overlap. PD with ICDs, Parkinson's disease with impulse control disorders. [file Image_4.tif]

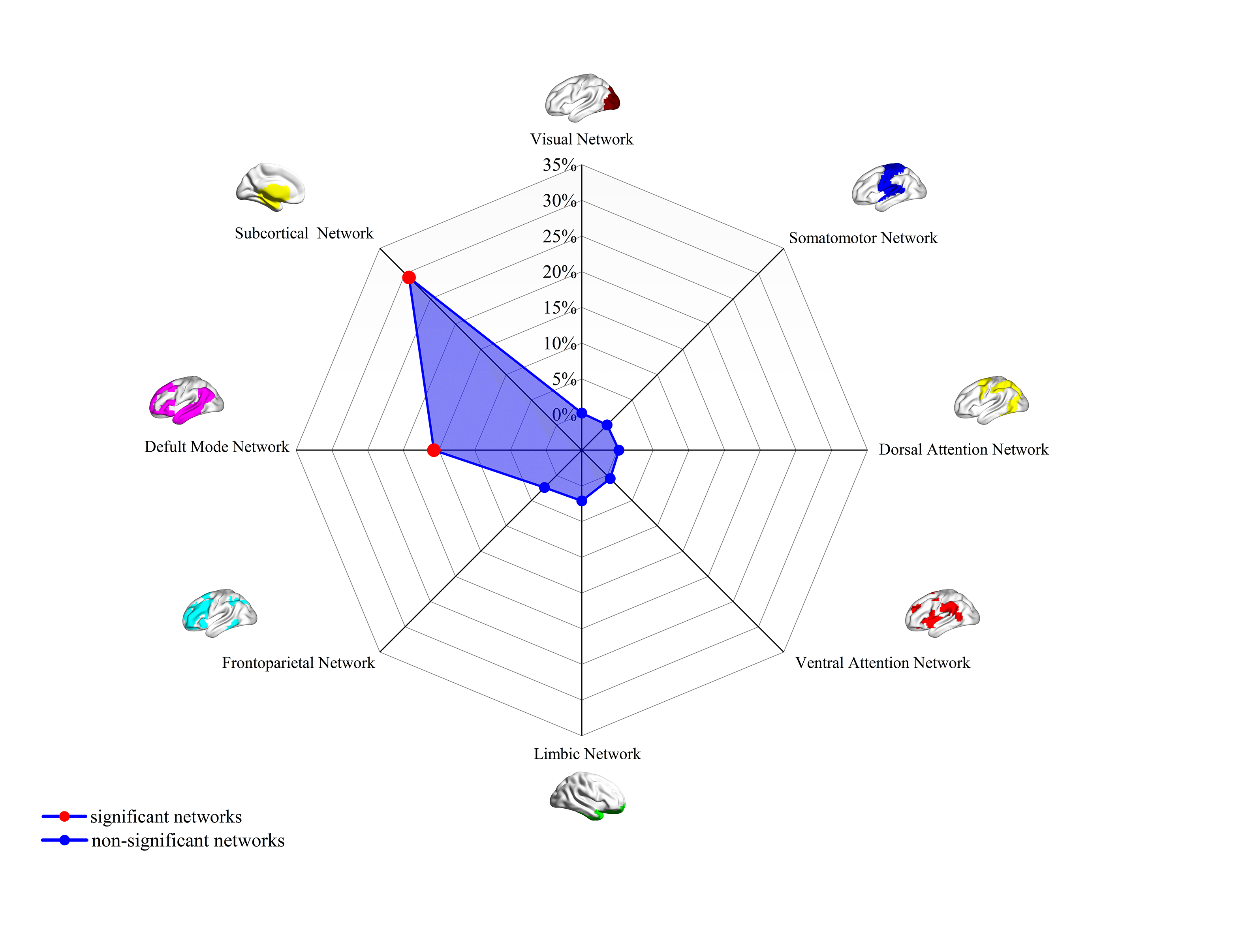

Supplement: Supplementary Figure S5 — Associations of dysfunctional brain networks with canonical brain networks in PD with ICDs based on 7-mm radius sphere. Polar plots display the proportion of overlapping voxels between each brain dysfunctional network and a canonical network relative to all voxels within the corresponding canonical network. The red circles represent brain dysfunction networks, defined as significant networks, exhibiting ≥ 10% overlap with canonical networks, whereas the blue circles represent non-significant networks with < 10% overlap. PD with ICDs, Parkinson's disease with impulse control disorders. [file Image_5.tif]
