# Supplementary material for: Neural network localization in Parkinson’s disease with impulse control disorders
Source: Front Aging Neurosci. 2025 Mar 28;17:1549589. doi: 10.3389/fnagi.2025.1549589 (PMC11985847; doi:10.3389/fnagi.2025.1549589)
Supplement: Supplementary file 6 [file Table_1.docx]

**Table S1.** Demographic information of the HCP

| **Dataset sample size** | **Age (years)** | **Gender (F/M)** |
| --- | --- | --- |
| HCP 1093 | 28.78±3.69 | 594/499 |

Age is expressed as mean ± standard deviation. Note: HCP, Human Connectome Project; F, female;

M, male.
