# Supplementary material for: Neural network localization in Parkinson’s disease with impulse control disorders
Source: Front Aging Neurosci. 2025 Mar 28;17:1549589. doi: 10.3389/fnagi.2025.1549589 (PMC11985847; doi:10.3389/fnagi.2025.1549589)
Supplement: Supplementary file 7 [file Table_2.docx]

**Table S2.** Resting-state fMRI parameters of the HCP

| **Parameter** | **HCP** |
| --- | --- |
| Scanner | 3.0T Siemens Trio |
| Sequence | GRE-EPI |
| TR (ms) | 720 |
| TE (ms) | 33.1 |
| FA (°) | 52 |
| FOV (mm²) | 208×180 |
| Matrix size | 104×90 |
| Slice thickness (mm) | 2 |
| Slice gap (mm) | 0 |
| Slices | 72 |
| Time points | 1210 |

HCP, Human Connectome Project; GRE-EPI, gradient-recalled echo-Planar Imaging; FA, flip angle; fMRI, functional magnetic resonance imaging; FOV, field of view; TE, echo time; TR, repetition time.
